# Supplementary material for: U.S. nursing home leadership experiences with COVID-19 and its impact on residents and staff: A qualitative analysis
Source: PLoS One. 2023 Dec 19;18(12):e0293336. doi: 10.1371/journal.pone.0293336 (PMC10729989; doi:10.1371/journal.pone.0293336)
Supplement: S1 Appendix — (DOCX) [file pone.0293336.s001.docx]

**S1 Appendix 1. Research Protocol**

| **Qualitative**  **Analysis Phase [1]** | **Research Stage** | **Action** | **Details** |
| --- | --- | --- | --- |
| Assemble Qualitative Analytic Team - *3 researchers* | Survey Development | Develop survey content | - 3 open-ended questions for textual response with instructions - Research team reviews and revises - consensus |
|  |  | Cognitive Interviews [2] –  pretest open-ended questions | - 4 subjects from target audience complete survey - Collect feedback (including understanding of survey content, ability to answer survey questions, etc.) - Revise, present to research team, and finalize |
|  | Develop Survey Sample | Construct a national sample stratified by size and quality rating | - Obtain list of all US nursing homes rated by the Centers for Medicare and Medicaid Services - Exclude those with <30 beds - Group homes by size and quality rating (6 groups: 30-99 beds x 3 levels of quality ratings* and 100+ beds x 3 levels of quality rankings*) - Select a national sample of 283 nursing homes in each group - Obtain names, email addresses and postal addresses for directors of nursing or administrators for each nursing home (search online, phone calls) |
|  | Survey Distribution and Collection | Send survey and collect data from national sample | - Paper surveys sent by postal service (include link to online survey)   - Postage paid return envelope - Online survey link sent via email   - - - - Data collection via Qualtrics https://www.qualtrics.com/ - Employ reminder protocol and distribute incentives |
|  |  | Compile qualitative textual responses | - Download textual responses for each qualitative question from Qualtrics   - Quality control: check for any truncated responses and correct - Manually transcribe responses on paper surveys   - Quality control: Independent reviewer checks transcriptions; areas of disagreement discussed and resolved - Assemble data into Excel spreadsheet and Word document.to prepare for analysis |
| 1 – Data Familiarization | Immersion Crystallization [3] | In-depth independent review of each response set | - Each analytic team member is assigned the response set for one of the open-ended questions. - Open reading and deep review |
| 2 – Data Categorization  3 – Theme Generation |  | Prepare independent summaries | - Each team member prepares a detailed summary of responses to their assigned question identifying component themes and associated quotes |
|  |  | In-depth independent review of a second response set - summaries | - Each team member is assigned a second open-ended question for independent open reading and deep review, preparation of a summary, and identification of component themes and associated quotes |
|  |  | Presentation and discussion of both summaries (with themes and associated quotes) | - For each open-ended question, both of the summaries are presented to the research group and discussed. - Individual team members can further develop or revise their summaries based on feedback - Individual summaries are finalized |
| 4 – Theme Development and Review | Consolidation of Summaries | Independent summaries are consolidated into a combined consensus summary (with themes and associated quotes) | - Each open-ended questions is assigned to the team member who has not prepared an independent summary - The team member reviews the response set for the open-ended question - Both previously prepared independent summaries are reviewed - The team member prepares a consolidated summary including themes and associated quotes. |
|  | Consensus | Consolidated summaries are reviewed and revised | - Presentation and review of each open-ended consolidated summary to the analysis group - Discuss and revise to reach consensus - Finalize consolidated summaries |
| 5 – Theme Refining, Defining, and Naming | Construct Combined Theme List | Construct a single list of themes from each of the consolidated summaries | - Review themes from across all 3 questions - Combine themes as needed - Organize into themes and subthemes - Construct a final theme list with associated quotes - Present the final theme list to the research team - Revise as needed to reach consensus - Identify cross-cutting themes that need to be further defined and refined |
|  |  | Code new cross-cutting themes and prepare summaries | - Define the cross-cutting themes - Code response excerpts from all response sets - Generate code reports - Two team members independently summarize each theme - Independent summaries are presented to the analytical team - Revise summaries and combine into consolidated summaries with associated quotes - Present consolidate summary to analytical team and revise as needed to reach consensus |
|  |  | Final theme list & associated quotes | - Assemble all themes and associated quotes into a single list. |
| 6 – Writing Up | Results | Prepare results section of manuscript | Prepare summary paragraphs for each theme  Identify 1-2 illustrative quotes for table of themes, subthemes and illustrative quotes |

* Quality Ratings = 0-5 stars; 3 Groups - 1 star, 2-4 stars, 5 stars

**References**

1. Braun V, Clarke V. Thematic Analysis: A Practical Guide. London: Sage Publications Ldt, 2022. ISBN 978-1-4739-5323-9

2. Willis, GB. Cognitive interviewing: A tool for improving questionnaire design. Thousand Oaks: Sage Publications, 2004. ISBN: 9780761928034

3. Borkan JM. Immersion-crystallization: a valuable analytic tool for healthcare research. Fam Pract. 2022. 39(4): 785-789. doi: 10.1093/fampra/cmab158.
