# Supplementary material for: U.S. nursing home leadership experiences with COVID-19 and its impact on residents and staff: A qualitative analysis
Source: PLoS One. 2023 Dec 19;18(12):e0293336. doi: 10.1371/journal.pone.0293336 (PMC10729989; doi:10.1371/journal.pone.0293336)
Supplement: Attachment — Submitted filename: Response to Reviewers.docx [file pone.0293336.s002.docx]

**Rebuttal – Response to Reviewers**

Responses are in **BOLD**.

Responses to Editor Comments

1. PLOS ONE's style requirements – **We have attempted to implement all style requirements as indicated.**

2. ETHICS -- Please provide additional details regarding participant consent. In the ethics statement in the Methods and online submission information, please ensure that you have specified what type you obtained (for instance, written or verbal, and if verbal, how it was documented and witnessed). If your study included minors, state whether you obtained consent from parents or guardians. If the need for consent was waived by the ethics committee, please include this information.

**Thank you for your careful consideration of subject consent. In this case, our Institutional Review board determined that the study posed minimal risk and was EXEMPT. Their determination was:**

**Human research that is exempt under the following guideline(s):**

**• 45 CFR 46.104(d)(2) Educational tests/survey/interview procedures, or observation of public behavior**.

**With exempt research, formal consent is not required. However, at the beginning of the survey we included the following statements:**

- **Please fill out this survey and return it in the postage-paid envelope.**
- **Your participation in this study is voluntary. If you come across a question you would rather not answer, feel free to skip it and go on to the next question.**
- **Your answers are confidential. No information will be presented or published in any way that would permit identification of any individual or facility. Your name and answers will not be shared with anyone other than the researchers.**
- **Thank you for taking the time to help with this very important project.**

**In the preamble to the open-ended questions used for this qualitative study, we also stated the following:**

**If you would like to tell us more about your COVID-19 experiences at your nursing home, please answer the following questions. If not, please return the survey in the enclosed envelope.**

**Please reflect upon how you, your facility, and direct care staff responded to resident loneliness and social isolation during the pandemic. We want to hear your candid thoughts and opinions. Your responses will not be linked to your name, but may be reported verbatim along with comments provided by other participants.**

**We consider voluntary completion of the survey to be evidence of consent and participants who did not consent simply did not fill out the survey. No further written or verbal documentation of consent was obtained or required by the UMass Chan or UMass Boston IRB. We have provided additional clarification in the Human Subjects section.**

Once you have amended this/these statement(s) in the Methods section of the manuscript, please add the same text to the “Ethics Statement” field of the submission form (via “Edit Submission”).

**We have added and clarified the Ethics Statement in the submission portal.**

3. Data Availability Important: If there are ethical or legal restrictions to sharing your data publicly, please explain these restrictions in detail. Please see our guidelines for more information on what we consider unacceptable restrictions to publicly sharing data: [http://journals.plos.org/plosone/s/data-availability#loc-unacceptable-data-access-restrictions](https://nam10.safelinks.protection.outlook.com/?url=http%3A%2F%2Fjournals.plos.org%2Fplosone%2Fs%2Fdata-availability%23loc-unacceptable-data-access-restrictions&data=05%7C01%7Ccatherine.dube%40umassmed.edu%7C76f7ae5967b84db4c87008db2b298596%7Cee9155fe2da34378a6c44405faf57b2e%7C0%7C0%7C638151229727411466%7CUnknown%7CTWFpbGZsb3d8eyJWIjoiMC4wLjAwMDAiLCJQIjoiV2luMzIiLCJBTiI6Ik1haWwiLCJXVCI6Mn0%3D%7C3000%7C%7C%7C&sdata=gGIZLkKjx1LW%2BvHIdjFbsn4srYS13qsg9VP6gNHP7Ls%3D&reserved=0). Note that it is not acceptable for the authors to be the sole named individuals responsible for ensuring data access.

Human research participant data and other sensitive data

For studies involving human research participant data or other sensitive data, we encourage authors to share de-identified or anonymized data. However, when data cannot be publicly shared, we allow authors to make their data sets available upon request.

If there are ethical or legal restrictions on sharing a sensitive data set, authors should provide the following information within their Data Availability Statement upon submission:

Explain the restrictions in detail (e.g., data contain potentially identifying or sensitive patient information)

**These qualitative data include highly personal information that could allow identification of participants. In order to obtain the most honest and unbiased responses, we promised responders the following:**

- **Your answers are confidential. No information will be presented or published in any way that would permit identification of any individual or facility. Your name and answers will not be shared with anyone other than the researchers.**

**It would be unethical to share our data when we assured our study participants that their data would confidential and that their answers would not be shared. Responses to open-ended questions can be quoted in our manuscript however, due to the statement printed in the preamble to the open-ended questions on our survey:**

**Your responses will not be linked to your name, but may be reported verbatim along with comments provided by other participants.**

**Data collection for this research was conducted in 2022 prior to US National Institute of Health requirements for data sharing had taken place. (https://sharing.nih.gov/data-management-and-sharing-policy/about-data-management-and-sharing-policies/data-management-and-sharing-policy-overview)**

Provide contact information for a data access committee, ethics committee, or other institutional body to which data requests may be sent

**If data is requested for reanalysis or confirmation of results, the petitioner would need to contact our ethics committee:**

**Center for Clinical and Translational Science**

**UMass Chan Institutional Review Board (IRB)**

**362 Plantation Street,**

**Ambulatory Care Center, 7th Floor**

**Worcester, MA 01605-0002**

**Phone: (508) 856-4261**

**IRB@umassmed.edu**

Response to Reviewers' comments:

Reviewer #1: Dear authors,
I am more than pleased to review this manuscript. Thank you for giving such great opportunity.
In general, the topic is interesting and adds a significant contribution to leadership and geriatrics sciences, however, the manuscript need major revisions, organized, and summarized.
Please follow the following comments;

**Thank you for your positive comments and your thorough review. We hope that our clarifications and responses satisfactorily address your concerns.**

Abstract:
- Research design should be clearly stated –  **We provided additional details about the survey design.**
- Number of nursing homes were data are collected must be mentioned – **Each respondent was from a different nursing home. We clarified – the number of respondents is the same as the number of nursing homes.**
- in setting and participants section : add home to directors of nursing – **Respondents are now called “nursing home directors of nursing” – these are the leaders of the clinical staff in US nursing homes and are referred to as “Directors of Nursing” or DON.**
- Results must be summarized – **Thank you. We reviewed this section and believe our results are summarized in the abstract..**
- No need to add quotes from participants' entries in abstract -- **The quote was removed.**

Introduction:
- Background is very weak, literature review need to be placed in the international context of the findings in relation to the chosen research problem **– We have added international context and revised the introduction.**

- It is better to avoid pronouns referring to authors (replace "we" in the end of introduction with "the current study"). – **We have removed the word “we” from the manuscript.**

Method:
This section include many details not needed and many important items are missing
- Qualitative design is not clear

- Reasons for choosing the qualitative design to address research problem of this study are not stated –

**The Methods section was revised. Importantly, the following information about the qualitative design was added:**

**Qualitative inquiry guided by interpretivist epistemology was employed to elicit the experiences of nursing home directors of nursing/administrators during the COVID-19 pandemic. This approach was selected to better understand participant perspectives through stories of personal, staff, and resident pandemic experiences. Stories about strategies employed to address social isolation during COVID-19 were also elicited. Textual responses to optional open-ended survey questions were employed to enhance data collection from a diverse national (U.S.) sample and to maximize personal expression, allowing participants wide latitude to describe strategies, experiences, and the overall impact of the COVID-19 pandemic in their own words. Open-ended questions were appended to a national (U.S.) nursing home survey and were marked “optional.” Open responses and confidentiality protections were intended to minimize response bias. This approach was selected for efficiency and expediency at a time when the COVID-19 pandemic was beginning to wind down and pandemic experiences were still fresh. An iterative reflexive thematic approach to analysis was employed.**

I think if it was conducted as mixed study it will give powerful quantitative impressions about effects on residents and staff, challenges, strategies to mitigate loneliness and social isolation, and benefits.

**Thank you for your suggestion. We chose to structure this as 2 studies from the outset (as opposed to a single mixed methods study) so that we would have ample space to present the results of each study in separate manuscripts. We intend to provide references to each of these studies within these manuscripts when citation information is available. An initial citation is provided. (*Lapane KL, Lim E, DS Mack, Hargraves JL, Cosenza C, Dubé CE. Rising to the occasion: a national nursing home study documenting attempts to address social isolation during the COVID-19 pandemic. JAMDA. Forthcoming.*)**

- Setting should be described enough ( I cannot find simple information as the total numbers of nursing homes where stratums are drawn)

**Thank you. We have included the total number of nursing homes. Additional information about the sample is provided in the section “Survey sample and process”.**

- Much emphasis must be given to participants ( total population; characteristics of participants in terms of job titles, roles in nursing homes; and recruitment measures)

**Participant characteristics including job titles are provided in Table 1. Characteristics of Participants. There was a small number (4%) of job titles that were neither Director of Nursing nor Nursing Home Administrator. Below Table 1 we have provided additional detail on “Other” job titles. Table 2 provides distribution of participating nursing homes by state. Additional information about the sample is provided in the section “Survey sample and process”.**

- Causes of low response rate (30%) must be discussed

**The National Nursing Workforce Survey 2020, estimated the US response rate among licensed nurses to be 32.6% . Our response rate is similar. [Smiley et al Journal of Nursing Regulation, April 2021, Volume 12, Issue 1 Supplement** [**https://www.journalofnursingregulation.com/article/S2155-8256(21)00027-2/fulltext**](https://www.journalofnursingregulation.com/article/S2155-8256(21)00027-2/fulltext) **]**

**We do acknowledge that our response rate may have been affected by the very factors described in many responses to our questions about pandemic experiences. The population we were targeting included nursing home leaders who had just experienced a very stressful and traumatic period of time, accompanied by additional reporting requirements due to state and federal COVID-19 regulations. This voluntary survey was yet another demand on their time, and it is feasible to believe that potential respondents were simply worn down from their clinical and administrative experiences and chose to focus their efforts elsewhere.**

**We have summarized the above information in the limitations section of the manuscript.**

- Reasons for excluding small nursing homes must be given

**Small nursing homes (<30 beds) were excluded because they differ significantly from larger nursing homes in terms of operations and resources. The average size of a US nursing home in 2022 was 106 beds. [https://www.kff.org/other/state-indicator/average-number-of-certified-nursing-facility-beds/?activeTab=map&currentTimeframe=0&selectedDistributions=average-number-of-certified-nursing-facility-beds&selectedRows=%7B%22wrapups%22:%7B%22united-states%22:%7B%7D%7D%7D&sortModel=%7B%22colId%22:%22Location%22,%22sort%22:%22asc%22%7D] – We have added this explanation.**

- What are the measures used to ensure validity and trustworthy of data collected from subjects?

**Nursing homes included in our sample were legitimate, Medicare evaluated facilities. Surveys were sent directly to nursing home leadership either by using their official email address or the address of the nursing home. Respondents were all professionals. We were seeking voluntary responses to open-ended questions at the end of the survey for this qualitative study, and thus qualitative respondents were self-selected from the larger group. We have provided a table of characteristics (Table 1) for comparison of open-ended question responders versus non-responders. As noted under Participant Characteristics, “Respondents to open-ended questions tended to be older, have a longer tenure at their current nursing home, and have more time in a leadership position than non-responders.”**

****All of the above information already appears in the manuscript.**

- Roles of researchers in qualitative analysis process are not needed in method section. **– We have removed these roles.**
- It is better to avoid pronouns referring to authors – **We have removed pronouns.**

Results:
- Too much details however significant results are not delineated

**We used a reflexive thematic qualitative analytic approach, qualitative results are descriptive, and there are no tests of significance. To highlight key results we have added a summary table (Table 4). To streamline the results section, we collected most supporting detailed quotes in Table 5.**

- It is better to give support to analysis using more quotes from participants self-reporting

**We have added a table of themes and illustrative quotes (Table 5).**

- Quantitative analysis if added in results it give significant value especially response s are perceptions

**Thank you. We agree with your statement – however as a qualitative study we focus on perceptions, opinions and personal experiences.**

Discussion:
- It does not follow guidelines of effective debate (results of similar and contradictory studies must be given)

**Thank you for this consideration. We were following PLOS guidance on Discussion sections (**[**https://plos.org/resource/how-to-write-conclusions/#:~:text=It%20should%20include%3A,your%20results%20and%20initial%20hypothesis**](https://plos.org/resource/how-to-write-conclusions/#:~:text=It%20should%20include%3A,your%20results%20and%20initial%20hypothesis)**. ) which defines the discussion section as “An effective discussion informs readers what can be learned from your experiment and provides context for the results.”**

1. **the results of your research,**
2. **a discussion of related research, and**
3. **a comparison between your results and initial hypothesis.**

**We have expanded “compare and contrast to previous studies”.**

- It is better to avoid pronouns referring to authors – **We have removed pronouns.**
- I feel that discussion does not add new than just results – **Please see #1 in PLOS ONE guidance above. We have expanded the “compare and contrast to previous studies” aspect of the discussion section.**

Conclusion:
- Is prefect however, it need to be focused and summarized –

**“Is prefect” – Do you mean perfect? If yes, thank you. We have revised the conclusion to be more focused.**

Implications:
- Are there no future research directions for this study? I think that perceptions of residents and staff will greatly add value than leaders **–**

**Thank you for your comment. We believe that further research into effective approaches to workforce development and retention is needed and have added this to the implications section. Other suggestions for additional research are noted in the Discussion section.**

Reviewer #2: Reviewer report:
Thank you for the opportunity to review the manuscript. The authors are suggested to apply the following comments to the manuscript:

INTRODUCTION
- Further support of published literature on the research topic nationally and internationally is required.

**Thank you. We have revised the introduction and updated the literature cited to provide additional international context.**

- A justification of why you used the survey as a data collection method is ultimately needed.

**Open-ended questions were added to collect stories and experiences from a diverse sample of nursing home directors of nursing or administrators from across the U.S.. This approach was chosen for efficiency and expediency as the COVID-19 pandemic was beginning to wind down and pandemic experiences were still fresh.**

**We have added this explanation to the Research Design section.**

MATERIALS AND METHODS
Survey sample:
- Please explain how the participants were selected.

**We had included the following information about participant selection in this section (Survey Sample and Process) but have expanded it. Please note that all survey participants were offered the opportunity to respond to open-ended questions:**

**First, we acquired a list of all US nursing homes rated by the Centers for Medicare & Medicaid Services (CMS) as of August 2021 was acquired. Small nursing homes with <30 beds were excluded. Nursing homes were stratified by size and CMS quality rating into six strata (30-99 or 100+ beds; 1, 2-4, or 5-star ratings) with 283 nursing homes randomly selected in each stratum. Directors of nursing for each nursing home were identified and contact information was collected. Links to the online survey were sent to those with email addresses. Paper surveys including a $5 incentive were mailed to all email non-responders and those without email addresses. An additional $40 was sent after survey completion.**

- Non-participation: Specify how many participants dropped out or refused to participate. Give the reason if applicable.

**This information was provided in the RESULTS section: “From an eligible sample of 1,676 nursing homes, 504 completed surveys were collected (response rate 30%) with 271 (54% of the 504 respondents) answering at least one open-ended question.” Responding to the survey was voluntary, and responses to open-ended questions were optional. We did not collect information from survey non-responders or reasons why those who did respond did not proceed to answer open-ended questions. Additional information about the characteristics of survey responders who did answer open-ended questions versus those who did not are provided in Table 1.**

The survey content:
- Explain more details on the first section as how it was developed, the total number of questions, and the number of questions for each topic

**Thank you. We included the total number of quantitative questions in the survey and additional information about topics covered. Further detail about survey questions are reported elsewhere. *(Lapane KL, Lim E, DS Mack, Hargraves JL, Cosenza C, Dubé CE. Rising to the occasion: a national nursing home study documenting attempts to address social isolation during the COVID-19 pandemic. JAMDA. Forthcoming.)***

- Describe how the survey was pretested before full implementation

**Thank you. We have provided a summary of our pretesting process used to revise/finalize the survey.**

- Lines 124:128 contain unnecessary details – **Thank you for your comment. We believe that transparency regarding limitations to responses is important. As a compromise, we shortened 2 sentences and deleted the others.**

Data collection/ survey process:
Explain the survey process separately after the materials.

**We have added a heading for the data collection section under “Survey content.”**

Data analysis
- It is better to include a thematic map to justify how the themes and the sub-themes are related.

**Thank you. We have provided a table with themes, subthemes and illustrative quotes to show these relationships. (Table 5)**

RESULTS:
- Tables: the illustrated data does not support the aim of the study. It's better to present the demographics of the sample only.

**We respectfully disagree with this comment. We believe that information about how those who responded to open-ended questions are the same or are different from those who did not is important and thus have included both sets of characteristics in Table 1. This information also addresses points raised by Reviewer 1. Other tables provide additional information about the sample, length of open-ended responses, and results. We contend that this information does indeed support the aim of the study.**

- Results need a major review as most finding need to be supported with participant quotations, in addition to reporting participant number.

**We have added participant numbers to all quotes in the results section and added Table 5 which includes illustrative quotes for each subtheme. Reviewer 1 called for streamlining the Results section – so we opted for a table of longer quotes instead of integrated quotes.**

DISCUSSION:
- Include the limitation of the study at the end of the discussion.

**Strengths and limitations were included at the end of the discussion section. (Second level heading.) We have expanded this section.**
REFERENCES:
- No 13: Is it unpublished work? It is unclear. – **This manuscript is currently forthcoming. We have updated the reference. (*Lapane KL, Lim E, DS Mack, Hargraves JL, Cosenza C, Dubé CE. Rising to the occasion: a national nursing home study documenting attempts to address social isolation during the COVID-19 pandemic. JAMDA. Forthcoming)***
- No 15: rewrite it "Lumivero - Software Solutions for Data Analysis & Management [Internet]. [cited 2022 oct 12]. Available from: <https://lumivero.com/>"

**Thank you. We have updated this reference as you suggest.**
