# Supplementary material for: U.S. nursing home leadership experiences with COVID-19 and its impact on residents and staff: A qualitative analysis
Source: PLoS One. 2023 Dec 19;18(12):e0293336. doi: 10.1371/journal.pone.0293336 (PMC10729989; doi:10.1371/journal.pone.0293336)
Supplement: Attachment — Submitted filename: Reviewer PONE-D-22-32109.docx [file pone.0293336.s003.docx]

Paper Title: *US Nursing Home Leadership Perceptions of COVID-19’s Impact on Residents and Staff: AQualitative Analysis*

Journal Manuscript ID: PONE-D-22-32109

**REVIEW**

This study wants to examine experiences of nursing home leadership addressing resident loneliness and social isolation during the COVID-19 pandemic in the US and personal and professional impacts on themselves and staff.

I think that the focus of this study could be important. Congratulations for your work and effort. However, I would like you to pay attention to the following indications for improvement its importance.

**Abstract:**

- Why does the abstract include the number of the participant who has said the sentence(“*The pandemic has been a heartbreaking experience, and we will be trying to recover as an industry for years to come*”), and the text, in the results section, does not do the same with the rest of the participants and their sentences?

**Introduction:**

Based on the information presented in the introduction, key information missing pertains to the following:

- The introduction is very weak. I need the following questions (that make up the introduction) to be answered in more depth: What is the problem? Why is it important? What is known so far (scientific articles)? What is not known? Link to the objectives of the study.

**Methodology**

Information provided in the methods and procedures is not detailed or clear enough to replicate the study. See below for suggestions/ feedback.  Also, we strongly recommend you to use the *Consolidated Criteria for Reporting Qualitative Research guidelines* and

*The Working Group on Journal Article Reporting Standards for Qualitative Research*.

- Please, begin this section with Theoretical framework: What methodological orientation was stated to underpin the study? e.g. grounded theory, discourse analysis, phenomenology, content analysis.
- Participant selection: in this section clarify the following issues and not in another section.
  - How were participants selected? e.g. purposive, convenience, consecutive, snowball.
  - How many participants were in the study? Sample size calculation.
  - How many people refused to participate or dropped out? Reasons?
- Setting: use this section to talk about Where was the data collected? e.g. home, clinic, work place; Was anyone else present besides the participants and researchers?; What are the important characteristics of the sample? e.g. demographic data, date.
- Add headings such as *Data analysis and rigor, Data collection.*

**Results**

- Please make the tables (at least table 1) smaller and more dignified by highlighting or bolding important headings, such as age, gender, etc.
- Table 2, make it smaller.
- Table 3, idem. Add it the standard derivation.

**Discusión**

- It would be good starting with the purpose of the study.
- In my opinion, there should be more scientific discussion with more references to scientific articles.
- Discuss the themes as you have placed them in the results.

**References**

- Only 22 references is too few.
- Review the bibliography guidelines according to the journal's standards. Not all of them are well referenced
