# Supplementary material for: U.S. nursing home leadership experiences with COVID-19 and its impact on residents and staff: A qualitative analysis
Source: PLoS One. 2023 Dec 19;18(12):e0293336. doi: 10.1371/journal.pone.0293336 (PMC10729989; doi:10.1371/journal.pone.0293336)
Supplement: Attachment — Submitted filename: Response to Reviewers.docx [file pone.0293336.s004.docx]

**Response to Reviewers**

Paper Title: *US Nursing Home Leadership Experiences with COVID-19 and Its Impact on Residents and Staff: A Qualitative Analysis*

Journal Manuscript ID: PONE-D-22-32109

**PLEASE NOTE: Responses are provided in BLUE**

4. Have the authors made all data underlying the findings in their manuscript fully available?

The [PLOS Data policy](https://nam10.safelinks.protection.outlook.com/?url=http%3A%2F%2Fwww.plosone.org%2Fstatic%2Fpolicies.action%23sharing&data=05%7C01%7Ccatherine.dube%40umassmed.edu%7C2423e5c1136b47e4a34608db80f65573%7Cee9155fe2da34378a6c44405faf57b2e%7C0%7C0%7C638245568061718587%7CUnknown%7CTWFpbGZsb3d8eyJWIjoiMC4wLjAwMDAiLCJQIjoiV2luMzIiLCJBTiI6Ik1haWwiLCJXVCI6Mn0%3D%7C3000%7C%7C%7C&sdata=NvRpUZz4y8vQqNOGvbjCNXTzeDHvLbubYJNYN1rCF1I%3D&reserved=0) requires authors to make all data underlying the findings described in their manuscript fully available without restriction, with rare exception (please refer to the Data Availability Statement in the manuscript PDF file). The data should be provided as part of the manuscript or its supporting information, or deposited to a public repository. For example, in addition to summary statistics, the data points behind means, medians and variance measures should be available. If there are restrictions on publicly sharing data—e.g. participant privacy or use of data from a third party—those must be specified.

Reviewer #1: No

Reviewer #2: Yes

Reviewer #3: (No Response)

**RESPONSE: It would be unethical to make our qualitative data available as we do not have IRB approval or consent from participants to do so. We have justified this multiple times. If our previously provided detailed justification is insufficient, we will withdraw and submit to another journal.**

1. If the authors have adequately addressed your comments raised in a previous round of review and you feel that this manuscript is now acceptable for publication, you may indicate that here to bypass the “Comments to the Author” section, enter your conflict of interest statement in the “Confidential to Editor” section, and submit your "Accept" recommendation.

**Reviewer #1: All comments have been addressed**

Reviewer #2: All comments have been addressed

Reviewer #3: (No Response)

**RESPONSE: Reviewer 1 indicates that all comments in the previous review round have been addressed. However, new requests for revisions (particularly for the Introduction and Discussion) are included below. Most of the previous review comments focused on these sections as well. We believe we have addressed the requested revisions and making further demands for additional revisions is unwarranted. We have, however, made a good faith effort to address Reviewer 1’s issues.**

Reviewer #1: Dear authors

Thanks a lot for this great effort appeared in this manuscript
Thanks again for your responses. These responses reflect professional researchers able to upgrade knowledge and practice.
Manuscript is already modified than before; however, literature review needs to be strengthed in terms of research gap, magnitude of the problem, and context of the study. Also, discussion needs to be strengthed.

**REVIEW**

This study wants to examine experiences of nursing home leadership addressing resident loneliness and social isolation during the COVID-19 pandemic in the US and personal and professional impacts on themselves and staff.

I think that the focus of this study could be important. Congratulations for your work and effort.

**Thank you. However, we are concerned about the lengthy time required for PLOS ONE reviews and revisions resulting in delay of publication in PLOS ONE or elsewhere and ultimately reducing the relevance and impact of our work.**

However, I would like you to pay attention to the following indications for improvement its importance.

**Abstract:**

- Why does the abstract include the number of the participant who has said the sentence(“*The pandemic has been a heartbreaking experience, and we will be trying to recover as an industry for years to come*”), and the text, in the results section, does not do the same with the rest of the participants and their sentences?

**RESPONSE: Participant numbers do appear for all quotes in the Results section. It is unclear what this comment is referring to.**

**Introduction:**

Based on the information presented in the introduction, key information missing pertains to the following:

- The introduction is very weak. I need the following questions (that make up the introduction) to be answered in more depth: What is the problem? Why is it important? What is known so far (scientific articles)? What is not known? Link to the objectives of the study.

**RESPONSE: Much of this information already appears in the Introduction. We affirm that we have stylistic differences with Reviewer 1 but are making a good faith effort to address Reviewer 1’s issues. What already appears in the introduction is printed in RED. Revisions are printed in BLUE.**

What is the problem?

- Nursing homes were a vector for COVID-19
- 1.3. million nursing home residents were affected
- Loneliness and social isolation have been associated with negative health outcomes in general and among older people in congregate care settings such as nursing homes
- Loneliness and isolation among nursing home residents were exacerbated by COVID-19
- Staff were severely affected both by both illness and working conditions
- Nursing homes lost staff at high levels and staff shortages continue to be a problem

Why is it important?

- National studies in the US addressing the actions taken by nursing home leadership to address social isolation and the personal and professional impact of the pandemic on nursing home leaders, residents, and their staff are lacking
- Our study intends to further the collective understanding of these issues that may be pertinent to future pandemic preparedness

What is known so far (scientific articles)?

Integrated within the Introduction:

- Nursing home leaders rapidly developed new solutions to deal with a drastically worsening situation
- evidence-based solutions were not available
- working conditions were difficult in nursing homes and there was substantial psychological impact on the workforce
- Loneliness and social isolation have been associated with negative health outcomes including all cause mortality

What is not known?

- only a few national studies in the U.S have documented how U.S. nursing homes pivoted to provide care
- only a few national studies in the U.S. have documented how leadership, staff, and residents were impacted by the COVID-19 pandemic

Link to the objectives of the study.

- Aims were to explore strategies employed to address resident loneliness and social isolation in U.S. nursing homes and to describe the personal and professional impact of the pandemic on nursing home leaders, facilities, and their staff from the perspective of directors of nursing or administrators.

**Methodology**

Information provided in the methods and procedures is not detailed or clear enough to replicate the study.

**RESPONSE: We respectfully disagree. We provide a lot of detail, some of which was removed in response to Reviewer 1 comments in the last review. (** “Roles of researchers in qualitative analysis process are not needed in method section.” [Reviewer 1, Initial Review]**)**

**In particular, we describe:**

**1 – Use of a national survey to obtain responses to 3 open-ended questions designed to elicit stories about strategies employed to address social isolation among nursing home residents during COVID-19 and how professional experiences with the COVID-19 pandemic affected the respondent.**

**2 – Participant selection/recruitment**

**3 – Data collection approach**

**4 – The wording of the open-ended questions** (we have provided additional clarification)

**5 – A general description of the other survey questions** (reference to survey manuscript provided)

**6 – Survey question testing procedure**

**7 – Data collection approach**

**8 – Qualitative analysis approach including: use of modified inductive thematic analysis; an iterative process of emersion/crystallization; preparation of a consensus summaries; preparation of theme lists; detailed coding of cross-cutting themes.**

**We did not intend to teach readers how to conduct qualitative research. Our assumption is that if someone had qualitative research skills, they could replicate our work. If they were lacking qualitative research skills, they would need to seek out training to obtain those skills prior to replicating our work.**

**** However – in the spirit of a good faith effort, we have added a RESEARCH PROTOCOL as supplementary material.**

See below for suggestions/ feedback.  Also, we strongly recommend you to use the *Consolidated Criteria for Reporting Qualitative Research guidelines* and

*The Working Group on Journal Article Reporting Standards for Qualitative Research*.

**We are very familiar with the COREQ checklist and used it in the conduct and reporting of this research. The APA article you refer to above is a good reference and helpful in preparing a manuscript for a qualitative study. As a group, we have published multiple qualitative articles over the years. The details of the APA guidance attempts to address all qualitative research, but there are many different kinds. In *this* exploratory qualitative research study, we are not generating theory. Rather, we are reporting on the experiences of a population of people who lived through an unprecedented crisis unique in modern times.**

- Please, begin this section with Theoretical framework

**RESPONSE: We have added a conceptual model for the phenomenon under study based on Rapaccinia, et. al. 2020. Although this was developed for industry, the stages mirror what we anticipated the nursing home experience to be.** (Rapaccini M, Saccani N, Kowalkowskic C, Paiolae M, Adrodegari F. Navigating disruptive crises through service-led growth: The impact of COVID-19 on Italian manufacturing firms. Industrial Marketing Management 2020; 88: 225–237.)

- What methodological orientation was stated to underpin the study? e.g. grounded theory, discourse analysis, phenomenology, content analysis.

**RESPONSE: As stated in our manuscript “This exploratory qualitative inquiry [is] guided by interpretivist epistemology” and “A modified inductive thematic analysis approach, guided by principles of experiential reflexive thematic analysis, was employed.” We did not employ grounded theory, discourse analysis, phenomenology, or content analysis. We employed INTERPRETIVIST EPISTEMOLOGY and THEMATIC ANALYSIS.**

Interpretivist Epistemology

*Kivunja C, Kuyini AB. Understanding and applying paradigms in educational contexts. International Journal of Higher Education. 2017; 6(5):26-41.*

*Thematic Analysis:*

*Braun V, Clarke V. Thematic Analysis: A Practical Guide. London: Sage Publications Ldt, 2022.*

*Guest G, MacQueen KM, Namey EE. Applied Thematic Analysis. Thousand Oaks CA: Sage Publications Inc., 2012.*

*Tolley EE, Ulin PR, Mack N, Robinson ET, Succop SM. Qualitative Methods in Public Health: A Field Guide for Applied Research, Second Edition. San Francisco CA: John Wiley & Sons, 2016.*

- Participant selection: in this section clarify the following issues and not in another section.

**We have renamed the “Survey Sample and Process” section “Participant Selection and Survey Process”**

- How were participants selected? e.g. purposive, convenience, consecutive, snowball.

**We have described this. We have added clarification that the national sample was purposive while the final qualitative sample was a convenience sample comprised of those from the national sample who voluntarily responded to open-ended questions. This convenience sample still had good national representation (see Table 2).**

- How many participants were in the study? Sample size calculation.

**There is no sample size calculation in qualitative research. Adequate sample size is determined by SATURATION. Depending on the study and the data collected, saturation may be achieved with as few as 15 participants or fewer. We address saturation at the beginning of the Results section – which is where that discussion belongs.**

**Although we consider the number of participants to be part of our Results, we have added the number (271) to this Participant Selection section as well. It also appears in Table 1.**

- How many people refused to participate or dropped out? Reasons?

**We have indicated that 271 of 504 survey responders completed the optional open-ended questions. We have no way of knowing why some people chose to skip these optional questions. No one dropped out.**

- Setting: use this section to talk about Where was the data collected? e.g. home, clinic, work place;

**We indicated that surveys were sent to work email or postal addresses. However, participants could have completed these surveys anywhere.**

Was anyone else present besides the participants and researchers?;

**The researchers were not in the room as survey responses were collected online and by US postal service. It is unknown if others were present besides the participant.**

What are the important characteristics of the sample? e.g. demographic data, date.

**Please see tables 1 and 2.**

- Add headings such as *Data analysis and rigor, Data collection*

**We already have these sections. The first is called “Qualitative analytic approach” to which we have added: “and Rigor” – the heading Data Collection appeared in the last version of the manuscript.**

**Results**

- Please make the tables (at least table 1) smaller and more dignified by highlighting or bolding important headings, such as age, gender, etc.

**RESPONSE: It is unclear what is meant by “more dignified” tables. Important headings, such as age, gender, etc. were already bolded. We indented the cells below each heading to allow the headings to stand out more.**

**We also combined some categories in table 1 to make it a little shorter.**

- Table 2, make it smaller.

**RESPONSE: This table shows the response distribution by US state. There are 50 US States so we cannot make the table smaller. We added columns to make the table more compact.**

- Table 3, idem. Add it the standard derivation.

**RESPONSE: This table is already small (3 rows by 5 columns – and one of the columns was something you asked us to add.)**

**We are assuming that what you meant in the second part of your comment was “Add standard deviations.” We have rechecked our calculations and updated this table. We have included standard deviations.**

**Discusión**

- It would be good starting with the purpose of the study.

**We have added the purpose to the beginning of the discussion section.**

- In my opinion, there should be more scientific discussion with more references to scientific articles.
- Discuss the themes as you have placed them in the results.

**RESPONSE: We have revised the Discussion section once again, adding reflections on the conceptual model, headings corresponding to the 3 major themes, and additional references to scientific literature. Currently there are 32 citations to published literature in the Discussion section.**

**References**

- Only 22 references is too few.

**RESPONSE: There were 51 references provided, not 22. There were 27 references in the Introduction alone. In this most recent revised version there are 61 total references.**

- Review the bibliography guidelines according to the journal's standards. Not all of them are well referenced

**RESPONSE: It is unclear what this comment means. All citations are in proper format according to the journal’s guidelines and include all bibliographic information derived primarily from PubMed. A PLOS ONE editor has reviewed the reference list, and only indicated concern for one article that contained no page numbers (there are no page numbers for that article – it is an on-line article and the citation was provided by the publisher with no page numbers).**

**One reference that was, until now, listed as “forthcoming” has been updated with a complete citation as this article (submitted to another journal after initial submission of this article to PLOS ONE) has been published and is now available online.**

**On journal guidelines previously referenced only doi number was required, however we have added PMCID and PMC numbers wherever they were available.**
